# The Role of Incretins in Obstructive Sleep Apnea “GLP1RAs and GLP1RA/GIPRAs in OSA”

**DOI:** 10.1111/1753-0407.70170

**Published:** 2025-11-20

**Authors:** Ana Lucia Fuentes, Gurleen Dhami, Jeremy Pettus, Praveen Akuthota, Atul Malhotra

**Affiliations:** ^1^ Department of Pulmonary, Critical Care, Sleep Medicine, and Physiology University of California San Diego California USA; ^2^ Department of Pharmacology University of California Santa Barbara California USA; ^3^ Department of Endocrinology University of California San Diego California USA

## Abstract

Mechanisms of action and effects of GLP‐1 receptor agonists and dual GLP‐1/GIP receptor agonists on multiple organ systems relevant to obstructive sleep apnea and cardiometabolic health.

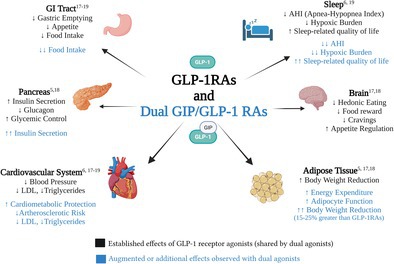

**Summary**

- This review examines the emerging role of incretin‐based therapies in the management of OSA.
- Evidence demonstrates that GLP‐1RAs and dual agonists lead to meaningful reductions in AHI and patient‐reported sleep outcomes.
- GLP‐1RAs and dual agonists represent a promising strategy for OSA, though long‐term use, comparative effectiveness with CPAP, and the impact in patients with diabetes remain important areas for future study.

## 1 Introduction

Obstructive Sleep Apnea (OSA) is a highly prevalent condition characterized by upper airway collapse during sleep, causing intermittent desaturations and arousals from sleep [1]. OSA affects nearly ~1 billion people worldwide [2] and is associated with hypertension, congestive heart failure, type 2 diabetes (T2DM), and cerebrovascular disease. Importantly, OSA affects up to 90% of patients with T2DM, and acts as a cumulative and synergistic risk factor for cardiovascular disease (CVD) [1, 2, 3, 4, 5]. Conventional treatments like continuous positive airway pressure (CPAP), oral appliances, and upper airway surgery are limited by patient adherence, variable treatment response and incomplete efficacy. Recently, pharmacotherapy has gained great interest, particularly regarding incretins which are gastrointestinal hormones crucial to metabolic regulation. Although incretins have been known for ~100 years, the emergence of glucagon‐like peptide 1 receptor agonists (GLP1RA) and glucose‐dependent insulinotropic polypeptide receptor agonists (GIPRA) has had a major impact. Tirzepatide, a dual GLP1RA/GIPRA, has proven effective in treating OSA and offers a promising addition to conventional therapies [6]. These pharmacotherapies may be especially beneficial in patients with T2DM, where metabolic and sleep‐related risks converge. Practitioners must be aware of the high burden of OSA, and the importance of timely diagnosis and management.

## 2 OSA in Diabetes Mellitus

The relationship between OSA and T2DM is multifaceted. OSA may contribute to insulin resistance through counter‐regulatory hormones released with repetitive apneas, though evidence that treatment of OSA improves glycemic control is inconclusive [7, 8, 9, 10]. Diabetes, in turn, may contribute to OSA through its effects on the control of breathing and/or pharyngeal neuromuscular function [11]. However, the role of diabetes‐related neuromyopathy in upper airway muscles remains uncertain. Chronic hyperglycemia can lead to autonomic and peripheral neuropathy, which may impair neural control of upper airway dilator muscles. The resulting reduction in muscle tone and delayed reflex activation during sleep increases pharyngeal collapsibility, offering a potential mechanistic link between diabetes and OSA. Clinical studies have demonstrated an association between diabetic autonomic neuropathy and greater OSA severity, supporting this relationship [12]. Beyond glycemic control, both OSA and T2DM independently increase CVD risk. Specifically, OSA impairs endothelial function, whereas T2DM affects vascular smooth muscle and microcirculation. These overlapping processes suggest that treating both conditions may be required to decrease vascular risk. While most data have focused on T2DM, OSA is also a concern in T1DM. Despite lower rates of obesity in T1DM compared to T2DM, these patients still demonstrate a high prevalence of OSA, with some cohorts reporting rates up to 46% even among those with a normal BMI [7, 8, 9, 10, 13]. These findings suggest that weight alone does not account for the high OSA burden. As in T2DM, OSA in T1DM independently predicts vascular disease. Additionally, patients with T1DM may be particularly sensitive to counter‐regulatory hormones due to their insulin deficiency and inability to compensate [7, 8, 13].

Although substantial data have shown improvement in both T2DM and body weight with GLP1RA, people with type 1 DM and T2DM were excluded from SURMOUNT‐OSA [6]. Because patients with T2DM experience less weight loss with GLP1RAs than those without T2DM, they may also derive less improvement in OSA from dual agonists like tirzepatide. Nevertheless, given their elevated vascular risk, patients with both OSA and T2DM may still derive substantial benefit. Similar questions remain in T1DM, where OSA is also common despite lower obesity rates, but data on treatment effects are lacking. Until more definitive evidence is available, clinicians should remain aware of these associations, which may already be clinically actionable.

## 3 Who to Screen

Given the high prevalence and clinical impact of OSA in DM, systematic screening is warranted. Patients who are obese, have central adiposity, resistant hypertension, or excessive daytime sleepiness are at particularly high risk and should be prioritized for evaluation. Other high‐yield groups include those with poor glycemic control despite adherence to therapy, recurrent nocturnal hypoxemia, or unexplained cardiovascular or microvascular complications. Traditional tools like the STOP‐BANG questionnaire and Berlin Questionnaire can aid in risk stratification, but their specificity is limited in patients with obesity and DM. Therefore, a low threshold for formal sleep evaluation is appropriate.

## 4 Current Treatment Landscape for OSA

Treatment is generally recommended upon diagnosis of moderate to severe OSA (defined as an apnea‐hypopnea index, AHI, ≥ 15/h) or for those with mild OSA and associated symptoms or underlying CVD [1, 2, 3]. In this manuscript, we will list here the therapeutic landscape prior to the addition of weight loss therapies such as GLP1/GIP receptor agonists. Current options include:

1. *Continuous Positive Airway Pressure (CPAP)*: CPAP is the first‐line treatment for OSA and is highly effective, reducing the apnea/hypopnea index (AHI) by up to 90% [4, 14]. By preventing airway collapse, CPAP restores airflow and sleep continuity yielding improved daytime sleepiness, quality of life, and mood. It is also associated with reductions in blood pressure, and may improve cardiovascular outcomes. However, real‐world effectiveness is limited by adherence, with adherence rates ~50%. Importantly, CPAP does not address underlying causes, such as obesity and may actually be associated with modest weight gain [12], highlighting the need for weight management in OSA patients.
2. *Oral Appliances*: Mandibular advancement devices (MADs) are a non‐invasive treatment option for mild‐to‐moderate OSA. These devices reposition the lower jaw and tongue, preventing airway collapse during sleep. Compared to CPAP, MADs yield smaller reductions in AHI, although they have similar improvements in daytime sleepiness, quality of life, and blood pressure reduction. Adherence is typically higher than CPAP (~60%–80%), which may narrow the effectiveness gap with CPAP [14]. However, MADs may be less effective in patients with severe obesity, large neck circumference, or severe OSA, and are therefore best suited for carefully selected patients who cannot tolerate CPAP.
3. *Upper Airway Surgery*: Upper airway surgery is a treatment option for patients with moderate–severe OSA who cannot tolerate CPAP. Surgical approaches include uvulopalatopharyngoplasty, multilevel pharyngeal procedures, tongue base reduction, maxillomandibular advancement, and tonsillectomy. Surgical treatments improve AHI and excessive sleepiness, with limited impact on blood pressure. Efficacy is variable, and surgery is less effective in patients with obesity (BMI > 35 kg/m^2^) or when non‐anatomic factors drive OSA.

Hypoglossal nerve stimulation (HGNS) is an additional type of surgery, in which an implanted device stimulates the hypoglossal nerve to control the tongue and prevent airway collapse during sleep. It has been approved as a treatment for moderate–severe OSA in CPAP‐intolerant patients. HGNS reduces AHI and improves daytime sleepiness, with the highest efficacy in patients with BMI < 32 kg/m^2^, and without complete concentric palatal collapse [15]. Randomized trials have been reported preliminarily but remain unpublished.

## 5 Weight Loss as Treatment of OSA

Weight loss is a key therapeutic strategy for OSA with two patterns of benefit [14, 15, 16]. Some suggest a threshold effect, where there is a substantial reduction in AHI once weight loss reaches a certain threshold. A meta‐analysis found that a 20% reduction in BMI corresponded to a ~57% decrease in AHI, with diminishing returns beyond that point. Others support a dose–response relationship, with one study reporting that each 1‐pound drop in weight, decreased AHI by ~1%.

When combined with CPAP, weight loss provides additional benefit to AHI reductions [14]. In the INTERAPNEA randomized trial [17], patients receiving CPAP plus an 8‐week lifestyle and weight‐loss program had AHI reductions of 51% at 8 weeks and 57% at 6 months, compared with 27% and 30% in the CPAP‐only group. This combined approach also yielded greater improvements in systolic blood pressure, body composition, and insulin resistance. Similarly, Chirinos [15] demonstrated that addressing both OSA and obesity together was more effective for reducing cardiometabolic risk than treating either condition alone.

Weight loss in patients with OSA can be achieved through lifestyle modification, bariatric surgery, and pharmacotherapy. Lifestyle interventions are often first‐line and consistently improve AHI and cardiometabolic health [15, 17, 18, 19], though long‐term weight loss and adherence to modifications are often limited. Bariatric surgery (e.g., gastric bypass, sleeve gastrectomy, etc.) leads to greater and more durable weight loss than lifestyle interventions alone, with improvements in AHI, daytime sleepiness, and cardiometabolic outcomes [15, 17, 18, 20, 21].

However, up to 45% of patients have residual OSA despite weight loss, and others may experience weight regain or fat redistribution that contributes to recurrence [18]. Residual disease is more likely in those with older age, high preoperative BMI or AHI, or non‐obesity factors.

## 6 GLP‐1 and GLP‐1/GIP Receptor Agonists: Mechanisms of Action

**FIGURE 1:**
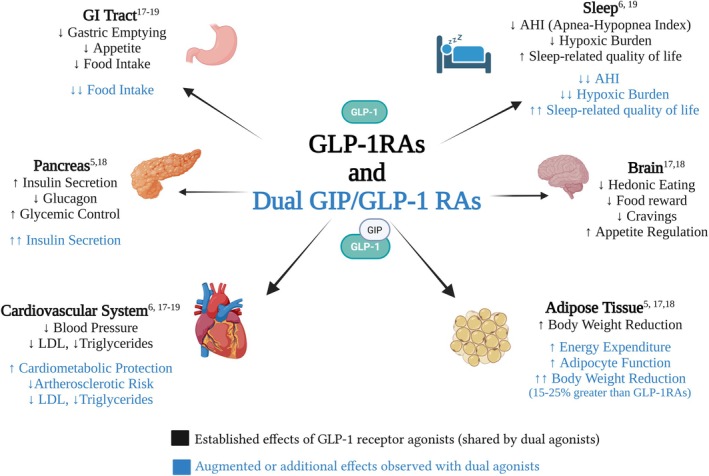
Mechanisms of action and effects of GLP‐1 receptor agonists and dual GLP‐1/GIP receptor agonists on multiple organ systems relevant to obstructive sleep apnea and cardiometabolic health.

Glucagon‐like peptide‐1 receptor agonist (GLP1RA) and GLP‐1/glucose‐dependent insulinotropic polypeptide (GIP) receptor agonists are promising therapies for OSA, largely through their effects on weight loss and metabolism [6, 20, 21, 22, 23]. GLP1RAs act centrally to reduce appetite through hypothalamic pathways and by dampening hedonic responses to food cues, leading to reduced intake and sustained weight loss. Dual agonists add GIP receptor activity, enhancing insulin secretion, improving adipocyte function, and increasing energy expenditure. Preclinical and clinical studies suggest that dual agonism produces greater reductions in food intake and body weight than GLP1RAs alone. In addition to these metabolic effects, dual agonists likely improve OSA through several mechanisms. As illustrated in Figure 1, their impact on glycemic control, insulin sensitivity, autonomic regulation, and central nervous system activity likely contribute to their observed benefits in OSA.

## 7 Clinical Evidence for GLP1RAs and GLP‐1/GIP Receptor Agonists

GLP1RAs such as semaglutide and dual agonists such as tirzepatide consistently produce substantial weight loss, a key driver of OSA improvement. In the STEP trials, semaglutide achieved sustained weight reductions [20], while the SURMOUNT trials showed even greater effects with tirzepatide (15%–22.5% weight loss), superior to semaglutide in head‐to‐head comparison (20.2% vs. 13.7% at 72 weeks) [21]. Both agents also improved cardiometabolic health, including blood pressure, lipids, and glucose regulation, with tirzepatide generally yielding greater benefits [6, 20, 21]. These findings highlight the potential of incretin‐based therapies as disease‐modifying treatments for OSA by targeting obesity and metabolic dysfunction.

### 7.1 Impact on OSA

In addition to the impact on weight loss and cardiometabolic outcomes, GLP1RA and dual agonists have shown efficacy in OSA. In the SCALE Sleep Apnea trial, adults with obesity and moderate to severe OSA who were unable to use CPAP received either liraglutide or placebo for OSA. Liraglutide led to a significant reduction in AHI, as well as increased weight loss over 32 weeks [22].

Building on this evidence, the SURMOUNT‐OSA trials included two parallel phase 3 randomized controlled trials evaluating tirzepatide in adults with obesity and moderate‐to‐severe OSA. SURMOUNT‐OSA trial 1 enrolled participants not using CPAP, whereas SURMOUNT‐OSA trial 2 enrolled participants adherent to CPAP. Across both trials (*n* = 469), participants were randomized to the maximally tolerated dose of tirzepatide (10 or 15 mg) subcutaneously or placebo for 52 weeks. In both trials, tirzepatide significantly reduced AHI (by ~25–30/h), and led to substantial weight loss (~18%–20%), with improvements in systolic blood pressure, sleep apnea specific hypoxic burden, inflammatory markers, and patient‐reported daytime and nighttime sleep outcomes [6].

The SURMOUNT‐OSA trials demonstrated important benefits of tirzepatide for OSA with obesity, but several limitations affect generalizability. Participants were limited to individuals with obesity (BMI ≥ 30 kg/m^2^) and moderate‐to‐severe OSA, excluding those with mild OSA (AHI 5–15/h), T2DM, or lower BMI ranges. The follow‐up period was 52 weeks, restricting conclusions about the long‐term durability of AHI reductions, long‐term effects on cardiovascular outcomes, and sustainability of weight loss. Data indicate that discontinuation of tirzepatide leads to significant weight regain, suggesting that its use for OSA may require prolonged or indefinite therapy [23]. However, the long‐term safety profile of tirzepatide with extended use remains unknown, and potential long‐term adverse effects warrant further investigation. Finally, SURMOUNT‐OSA did not compare active treatments e.g., tirzepatide vs. CPAP, limiting insights into comparative effectiveness. Future studies should evaluate whether combining tirzepatide with PAP therapy yields additive benefits.

Nevertheless, SURMOUNT‐OSA provides the first high‐quality evidence that pharmacologic therapy can meaningfully reduce OSA severity, opening the door to new disease‐modifying strategies. These findings highlight the potential of incretin‐based therapies to complement or extend beyond conventional approaches, particularly for patients with obesity‐related OSA who struggle with CPAP. Given the strong association between T2DM and OSA, future studies are clearly needed to define the role of these agents in patients with T2DM, where the potential benefits for both metabolic and sleep outcomes may be greatest.

## 8 Future Directions

Future research should aim to address several issues. Long‐term randomized controlled trials are needed to evaluate whether tirzepatide‐induced improvements in OSA are widely generalizable and translate into reductions in validated cardiovascular outcomes and durable AHI improvements beyond 1 year. Comparative effectiveness trials directly assessing tirzepatide, CPAP, and combination therapy could define optimal treatment strategies. Mechanistic studies integrating imaging, upper airway physiology, and biomarker assessments may help elucidate causal pathways. Given concerns about sarcopenia, future studies could address methods to limit loss of skeletal muscle using resistance exercise, altering protein intake, androgen modulators and/or medications such as bimagrumab.

## 9 Conclusion

GLP1RAs and dual receptor agonists represent a highly promising approach to OSA management, addressing both the underlying metabolic drivers of the disease and upper airway obstruction. Evidence demonstrates that pharmacologic weight loss can lead to clinically meaningful reductions in AHI and improved patient‐reported outcomes.

## Author Contributions

All authors contributed significantly to this work in accordance with the guidelines of the International Committee of Medical Journal Editors (ICMJE). **Ana Lucia Fuentes:** conceptualization, literature review, drafting of the manuscript, and critical revision for intellectual content. **Gurleen Dhami:** literature review, data organization, and drafting of manuscript sections. **Jeremy Pettus:** conceptualization, subject‐matter expertise in incretin‐based therapies, and critical revision of the manuscript. **Praveen Akuthota:** conceptualization and critical revision of the manuscript. **Atul Malhotra:** conceptualization, supervision, subject‐matter expertise in sleep and OSA, critical revision of the manuscript, and overall project oversight.

## Conflicts of Interest

The authors declare no conflicts of interest.

## Data Availability

Data sharing not applicable to this article as no datasets were generated or analyzed during the current study.
